# TFDP3 confers chemoresistance in minimal residual disease within childhood T-cell acute lymphoblastic leukemia

**DOI:** 10.18632/oncotarget.13630

**Published:** 2016-11-26

**Authors:** Ming Chu, Kailin Yin, Yujun Dong, Pingzhang Wang, Yun Xue, Peng Zhou, Yuqi Wang, Yuedan Wang

**Affiliations:** ^1^ Department of Immunology, School of Basic Medical Sciences, Peking University, Beijing, China; ^2^ Key Laboratory of Medical Immunology, Ministry of Health, Beijing, China; ^3^ Department of Hematology, Peking University First Hospital, Beijing, China; ^4^ Department of Anesthesiology, Fuling Center Hospital of Chongqing City, Chongqing, China

**Keywords:** TFDP3, E2F1, chemoresistance, minimal residual disease, childhood T-ALL

## Abstract

Acquired drug resistance in childhood T-cell acute lymphoblastic leukemia (T-ALL) remains a significant clinical problem. In this study, a novel gene therapy target for childhood T-ALL to overcome chemoresistance was discovered: *TFDP3* increased in the minimal residual disease (MRD) positive childhood T-ALL patients. Then, we established a preclinical model of resistance to induction therapy to examine the functional relevance of TFDP3 to chemoresistance in MRD derived from Jurkat/E6-1. Jurkat xenografts in NOD/SCID mice were exposed to a four drug combination (VXLD) of vincristine (VCR), dexamethasone (DEX), L-asparaginase (L-asp) and daunorubicin (DNR). During the 4-week VXLD treatment, the level of *TFDP3* increased 4-fold. High expression of TFDP3 was identified in the re-emerging lines (Jurkat/MRD) with increased chemoresistance, which is correlated with partially promoter demethylation of *TFDP3*. Downregulation of *TFDP3* by RNA interference reversed chemoresistance in Jurkat/MRD accompanied by reinstated E2F1 activity that coincided with increased levels of p53, p73, and associated proapoptotic target genes. Importantly, TFDP3 silencing *in vivo* induced apparent benefit to overcome chemoresistance in combination with VXLD treatment. Collectively, TFDP3 confers chemoresistance in MRD within childhood T-ALL, indicating that TFDP3 is a potential gene therapy target for residual cancer.

## INTRODUCTION

Acute lymphoblastic leukemia (ALL) is the most common childhood malignancy, and the most frequent cause of death from cancer before 20 years of age [1, 2]. T-cell ALL (T-ALL) accounts for 10% to 15% of childhood ALL, and constitutes up to 48% of high-risk patients [3, 4]. Recent advances in the treatment of childhood T-ALL have improved the 5-year survival rate above 75%, which is due to the intensive combination chemotherapies [5, 6]. The basic components of various therapies for children with T-ALL is comprised of an initial induction therapy (4-6 weeks) followed by intensive combination chemotherapy (6-8 months) and low-intensity “anti-metabolite” based maintenance therapy (18-30 months) [1]. Typically, induction therapy includes vincristine (VCR), dexamethasone (DEX), *L-*asparaginase (L-asp), and an optional use of an anthracycline such as daunorubicin (DNR) [1]. Despite the induction therapy resulting in complete remission rates of >95%, up to 25% of patients relapse and experience a 30% to 50% likelihood of survival [7]. Early relapse of childhood T-ALL remains the major cause of treatment failure, and suggests rapidly acquired resistance to multiple drugs [8].

Recently, the prognostic impact of minimal residual disease (MRD), which constitutes the most sensitive predictive factor for relapse in childhood T-ALL, has been extensively studied [9]. Patients with high MRD levels in bone marrow aspirates at the end of remission induction therapy have an increased risk of relapse, suggesting the presence of drug resistant subclonal populations within the MRD selected by specific chemotherapy schedules [10]. As most chemotherapeutic drugs are DNA damaging agents, which lead to a programmed cell death, defective apoptosis allows survival of these cells, making them resistant [11].

In 2007, we first reported the identification of a new member of DP family, human TFDP3, which was initially isolated as a novel cancer-testis antigen (CTA) in our screening for tumor-associated antigens [12–15]. Consistently, our results revealed that TFDP3 inhibits E2F1-induced, p53-mediated apoptosis in DNA-damaged cells *via* binding to E2F1 as a non-DNA-binding E2F1/TFDP3 complex [16]. Increasing evidences further confirmed that TFDP3 is a negative regulator of E2F1-induced cell death during DNA damage response [17–19]. In this study, we established a preclinical model of drug resistance to induction therapy in childhood T-ALL to broaden our understanding of TFDP3 function in chemotherapy resistance. Our data showed that TFDP3 confers chemoresistance in MRD within childhood T-ALL, indicating that targeting TFDP3 is a potential strategy for overcoming chemoresistance in minimal residual cancer cells, and stop progression to relapse and metastatic disease.

## RESULTS

### Association between *TFDP3* expression and MRD load in childhood T-ALL patients

**Figure 1:**
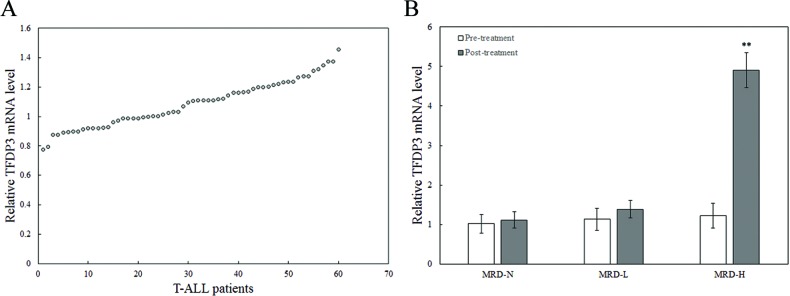
The expression of *TFDP3* in the MNCs from T-ALL childhood patients at diagnosis **A.** Relative expression of *TFDP3* in the MNCs from the 60 childhood T-ALL patients at diagnosis. **B.** Relative expression of *TFDP3* in the MNCs from the 60 childhood T-ALL patients at pre-treatment and post-treatment. The 60 childhood T-ALL patients were divided into three groups, including MRD-N, MRD-L and MRD-H. Relative *TFDP3* mRNA levels from T-ALL patients normalized to T cells from 5 healthy donors were analyzed by real-time PCR. Data are represented as mean±SD. ** Significance was determined at p<0.01 compared with pre-treatment.

To assess the expression status of *TFDP3* in childhood T-ALL, we determined the mRNA levels of *TFDP3* in the mononuclear cells (MNCs) from 60 T-ALL childhood patients at diagnosis (Table 1). The first group consisted of patients with undetectable MRD loads (MRD-negative), the second group had detectable MRD loads (<10^−4^ leukemic cells; MRD-low), and the third group consisted of MRD-positive patients with ≥10^−4^ leukemic cells (MRD-high) [9]. The entire group of patients showed low *TFDP3* mRNA levels before chemotherapy (Figure 1A). It is noteworthy that, at the end of remission induction therapy, the level of *TFDP3* was related to a 4-fold increase in the MRD-high (MRD-H) group in comparison with pre-treatment, whereas no significant changes were found in the MRD-negative (MRD-N) and MRD-low (MRD-L) group (Figure 1B).

**Table 1:** Clinical and biological features of pediatric T-ALL patients included in the study

Characteristics	MRD-negative patients (n=20)		MRD-low patients (n=20)		MRD-high patients (n=20)	
	No.	%	No.	%	No.	%
Sex						
Female	10	50	10	50	10	50
Male	10	50	10	50	10	50
Age, years						
< 10	12	60	11	55	14	70
10-17	8	40	9	45	6	30
Immunophenotype						
Early T	11	55	13	65	13	65
Other	9	45	7	35	7	35

### *In vivo* selection of childhood T-ALL xenograft lines

**Figure 2:**
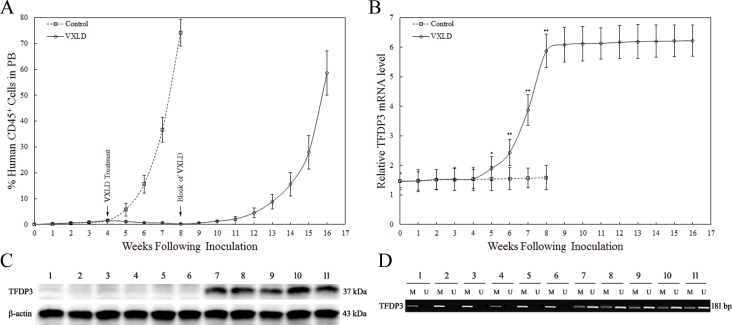
The expression of *TFDP3* during the murine T-ALL progression **A.** *In vivo* drug treatment of Jurkat xenografts. Jurkat xenografts were treated with a 4-week induction schedule of VXLD treatment, or saline (control), and then adopted an 8-week block of VXLD to *in vivo* selection of Jurkat/MRD and Jurkat/Control lines. **B.** The expression of *TFDP3* in the human CD45^+^ leukemia cells from murine PB. The relative *TFDP3* mRNA levels normalized to T cells from healthy donors were analyzed by real-time PCR (qPCR). Data are represented as mean±SD. ** Significance was determined at p<0.01 when compared with the control. **C.** The expression of TFDP3 protein in the harvest Jurkat-xenograft cell lines. Protein extraction obtained from Jurkat/E6-1 (1), Jurkat/Control (2-6) and Jurkat/MRD (7-11) were subjected to Western blotting using antibodies against TFDP3 and β-actin. **D.** The methylation status of CPG island of *TFDP3* promoter region. The DNA extracted from Jurkat/E6-1 (1), Jurkat/Control (2-6) and Jurkat/MRD (7-11) were subjected to methylation-specific PCR. The methylated (M) and unmethylated (U) bands were shown.

Here, we adopted a pre-clinical model of resistance to induction therapy in childhood T-ALL for *in vivo* selection of xenograft lines in the nonobese diabetic/severe combined immunodeficient (NOD/SCID) mice [20, 21]. Flow cytometric analysis of the NOD/SCID mice engrafted with Jurkat/E6-1 showed that the proportion of human *versus* murine CD45^+^ (%huCD45^+^) reached 1% at 4 weeks after inoculation (Figure 2A). Analogous to the clinical regimen, we optimized a 4-week induction schedule of VCR, DEX, ASP and DNR (VXLD), which clearly delayed disease progression in all Jurkat xenografts (Figure 2A). To allow T-ALL reappearance in the murine peripheral blood (PB), we developed a protocol consisting of an 8-week block of VXLD treatment. At harvest, %huCD45^+^ routinely exceeded 50% with spleen murine CD45^+^ cells being replaced with re-emerging cells (Jurkat/MRD). Jurkat/MRD lines were then purified and sorted using FITC-conjugated antihuman CD45 antibody on a FACSCalibur flow cytometer. Passage-matched controls (Jurkat/Control) were also harvested for each xenograft line. To confirm that the Jurkat/MRD and Jurkat/Control lines were derived from the corresponding Jurkat, Clone E6-1 (Jurkat/E6-1), short tandem repeat (STR) profiling, including D5S818, D13S317, D7S820, D16S539, vWA, TH01, AMEL, TPOX and CSF1PO, was performed and compared with Jurkat/E6-1 in the ATCC database. As shown in the Supplementary Table S1, the nine STR sites from Jurkat/MRD and Jurkat/Control lines completely matched the corresponding Jurkat/E6-1.

To investigate the functional relevance of induction therapy to TFDP3, we monitored the expression of *TFDP3* during the murine T-ALL progression. No significant change was found in the first 4 weeks, whereas during the following 4-week induction schedule of VXLD treatment, the %huCD45^+^ decreased along with increasing levels of *TFDP3*, and *A20* (known as TNFα-induced protein 3) (Figure 2B, Supplementary Figure S1). It is noteworthy that, at the end of the 4-week VXLD treatment, the level of *TFDP3* was related to a 4-fold increase, with no significant changes in the last 8-week block of VXLD treatment (Figure 2B). Thus, re-emerging lines were harvested and cultured in the absence of chemotherapy for 4 weeks, and then analyzed for TFDP3 protein expression using Western Blot. TFDP3 expression increased in Jurkat/MRD compared with Jurkat/E6-1 and Jurkat/Control (Figure 2C). In addition, the presence of TFDP3 promoter hypermethylation in Jurkat/E6-1 and Jurkat/Control was identified by methylation-specific PCR, whereas it was partially unmethylated in Jurkat/MRD lines (Figure 2D).

### TFDP3 confers multi-drug resistance to chemotherapeutic drugs

**Figure 3:**
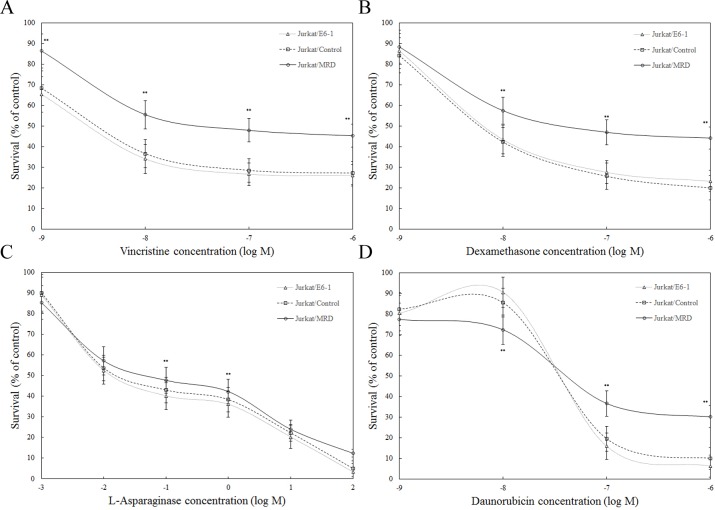
*Ex vivo* response of Jurkat xenograft lines to chemotherapeutic agents Parental and *in vivo* derived Jurkat xenografts were assessed using the MTT assay. *Ex vivo* response of Jurkat/E6-1, Jurkat/Control, Jurkat/MRD to vincristine **A.** dexamethasone **B.** *L-*asparaginase **C.** and daunorubicin **D.** was shown. Data represent the summary of five individual xenograft lines in Jurkat/Control and Jurkat/MRD lines. Three replicate experiments were performed in each case. Data are represented as mean±SD. ** Significance was determined at p<0.01 when compared with Jurkat/E6-1.

In recent clinical trials, patients with high MRD levels show an increased risk of relapse, suggesting the presence of multi-drug resistant (MDR) subclonals within the MRD stratum [10]. Cells isolated from *in vivo* drug-selected xenografts were tested *in vitro* against each of the four drugs in the induction schedule of VXLD treatment. Jurkat/MRD lines demonstrated increased resistance to VCR, DEX, ASP and DNR, whereas no acquired resistance was observed in Jurkat/Control, compared with Jurkat/E6-1 (Figure 3).

**Figure 4:**
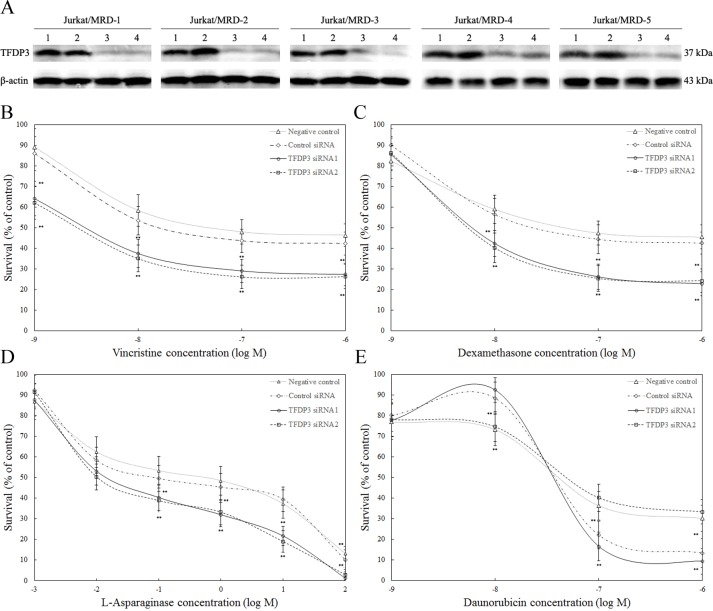
TFDP3 knockdown reversed the MDR phenotypes in the Jurkat/MRD cell lines **A.** Silencing of TFDP3 in Jurkat/MRD lines. Jurkat/MRD were transfected with control siRNA (2), *TFDP3* siRNA1 (3) or *TFDP3* siRNA2 (4). Non-transfection was used as negative control (1). Jurkat/MRD lines were treated with vincristine **B.** dexamethasone **C.** *L-*asparaginase **D.** and daunorubicin **E.** following TFDP3 knockdown, and assessed using the MTT assay. Data represent the summary of five individual Jurkat/MRD lines. Three replicate experiments were performed in each case. Data are represented as mean±SD. ** Significance was determined at p<0.01 when compared with the negative control.

To determine the functional relevance of TFDP3 to MDR pattern in MRD, Jurkat/MRD lines were transfected with *TFDP3* siRNA1 or siRNA2 to knock down TFDP3 and then treated with chemotherapeutic agents. Knockdown of TFDP3 were confirmed by Western blotting (Figure 4A). No activation of apoptosis was observed in TFDP3 knockdown Jurkat/MRD lines (Supplementary Figure S2). It is noted that suppression of TFDP3 reversed the MDR phenotype in Jurkat/MRD, resulting in increased apoptosis in response to each of the four drugs in VXLD (Figure 4B–4E).

### TFDP3 inhibits E2F1-mediated transcriptional activation

**Figure 5:**
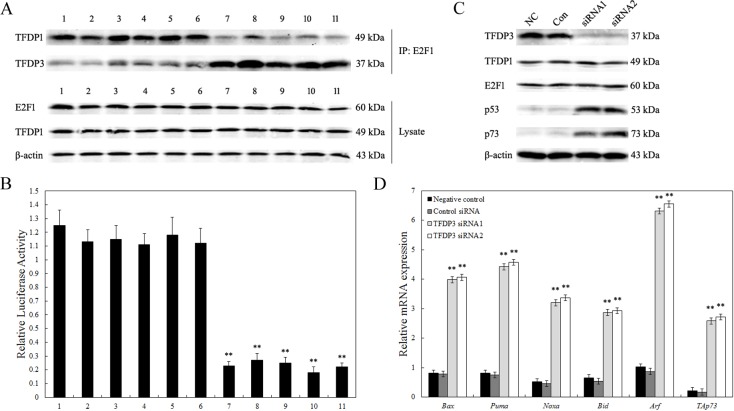
Inhibition of endogenous E2F1 transcriptional activity **A.** Co-immunoprecipitation for interaction of E2F1 with TFDP1 and TFDP3 intracellular. The cell lysates obtained from Jurkat/E6-1 (1), Jurkat/Control (2-6) and Jurkat/MRD (7-11) were analyzed. *Top*, cell lysate was immunoprecipitated with anti-E2F1, and the blot was probed with anti-TFDP1 and anti-TFDP3; *bottom*, cell lysate was analyzed for E2F1, TFDP1 and β-actin, respectively. **B.** Endogenous E2F1 transcriptional activity. Cells were transfected with 0.14 μg of E2F1 luciferase reporter plasmid and 0.14 μg of pRL-SV140 *Renilla* luciferase reporter plasmid. The cell lysates obtained from Jurkat/E6-1 (1), Jurkat/Control (2-6) and Jurkat/MRD (7-11) were analyzed. Data shown are averages of three independent experiments. ** p<0.01 when compared with Jurkat/E6-1 lines. **C.** Western blot analysis of p53 and p73. Jurkat/MRD-1 line was transfected with control siRNA (Con), *TFDP3* siRNA1 or *TFDP3* siRNA2. Non-transfected cells were used as the negative control (NC). **D.** Expression of E2F1 dependent genes. Relative expression of the E2F1 related proapoptotic genes was evaluated by qPCR in triplicate. ** Significance was determined at p<0.01 when compared with the negative control.

Our previous experiments have demonstrated that, as a member of the DP family, TFDP3 can compete with endogenous TFDP1 to form inactive complexes with E2F1 proteins [15]. As shown in Figure 5A, no change of E2F1 and TFDP1 expression level was observed in Jurkat/MRD, when compared to Jurkat/Control. Thus, E2F1 was immunoprecipitated from the cell lysates with anti-E2F1 antibodies, and the precipitates were then probed with anti-TFDP1 and anti-TFDP3 antibodies. TFDP3 was observed to compete with TFDP1 for E2F1 in Jurkat/MRD lines (Figure 5A). Is endogenous E2F1 activity affected by TFDP3? Indeed, we found that the base-line level of E2F1 activity in Jurkat/MRD with high expression of TFDP3 was significantly inhibited compared with Jurkat/E6-1 and Jurkat/Control using a luciferase reporter containing the E2F1 responsive element (Figure 5B).

As E2F1 is known to induce p53/p73-dependent apoptosis, we gain our insights into the molecular mechanisms underlying the TFDP3-mediated inhibition of E2F1-induced cell death. Figure 5C showed p53 and p73 increased in Jurkat/MRD-1 following TFDP3 knockdown. In view of the elevated p53 and p73, we further examined the mRNA levels of a select subset of p53/p73 target proapoptotic genes. *Bax, Puma, Noxa, Bid, Arf* and *TAp73*, which act in p53- and p73-dependent apoptosis, were markedly induced in the *TFDP3* silencing Jurkat/MRD-1 line (Figure 5D). These results reveal that knockdown of TFDP3 might reinstate E2F1 activity so as to reverse the MRD phenotype in Jurkat/MRD lines.

### *In vivo* efficacy of TFDP3 silencing in the treatment of T-ALL xenografts

**Figure 6:**
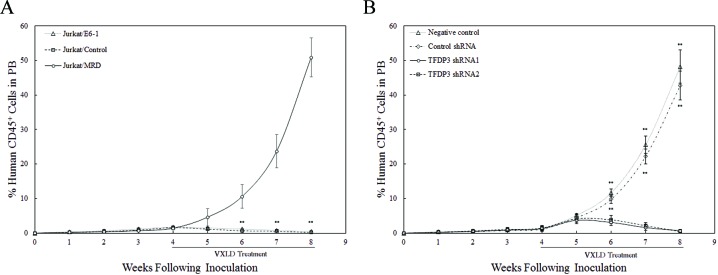
Efficacy of TFDP3 silencing for treatment of Jurkat xenografts **A.** Engraftment of Jurkat xenografts in NOD/SCID mice. Mice were inoculated with Jurkat/E6-1, Jurkat/Control, and Jurkat/MRD lines, and treated with a 4-week VXLD. During the progression of murine T-ALL, the leukemic burden was monitored by estimating the %huCD45^+^ in murine PB. ** Significance was determined at p<0.01 when compared with Jurkat/E6-1 lines. **B.** *In vivo* synergy effect of TFDP3 shRNAs in combination with VXLD treatment. Jurkat/MRD xenografts were treated with a 4-week VXLD, and assigned to receive intravenous either solvent (negative control) or non-effective shRNA (Control shRNA) or TFDP3 shRNA1 or TFDP3 shRNA2 twice per week. The %huCD45^+^ was monitored during the treatment. ** Significance was determined at p<0.01 when compared with the negative control.

Experiments were performed to assess whether the *in vitro* response to VXLD treatment reflected the *in vivo* experience of xenograft lines. Notably, Jurkat/MRD lines essentially grew through the VXLD treatment with the %huCD45^+^ routinely exceeded 50% at 8 weeks, whereas Jurkat/E6-1 and Jurkat/Control lines showed no acquired drug resistance to the VXLD treatment (Figure 6A). To determine *in vivo* efficacy of TFDP3 silencing in the VXLD treatment of T-ALL xenografts, we tested synergy of TFDP3 shRNA1 or shRNA2 in combination with VXLD in Jurkat/MRD. TFDP3 shRNAs alone induced no delay of Jurkat/MRD engraftment (Supplementary Figure S3). As shown in Figure 6B, the combination of TFDP3 shRNAs with VXLD induced apparent benefit to overcome MDR in Jurkat/MRD xenografts, indicating that TFDP3 silencing effectively reversed the *in vivo* MDR phenotype in Jurkat/MRD lines.

## DISCUSSION

Childhood T-ALL patients have a higher percentage of induction therapy failure, rate of relapse, when compared with the majority of ALL patients. The risk of treatment failure is 3 to 5 times as high among children with levels of MRD that are 0.01% or higher at the end of induction therapy than among those with levels that are lower than 0.01% [22, 23]. The presence of surviving MRD immediately after primary induction therapy suggests that they must be protected by some forms of genetic changes that are selected for through selective pressures imposed by chemotherapeutic drugs when these changes offer a survival advantage. Surviving foci of MRD can further develop permanent acquired MDR as a result of sequential genetic changes in response to the selective pressure of therapy [24]. Changes in expression of genes representing key biologic processes after induction therapy, and prognostic implication in MRD.

In 2007, we have characterized a novel cancer testis antigen, TFDP3 [15]. As a new member of the DP family, TFDP3 shares a high degree of sequence homology with TFDP1, which functions to enhance the DNA binding and the transcriptional activities of E2F1. Nevertheless, TFDP3 acts as an endogenous negative regulator for E2F1, which competes with TFDP1 for E2F1 binding, leading to the formation of inactive heterodimers and hence the inhibition of E2F1-induced cell death during the DNA damage [16, 17]. With a view to these features, we propose a model in which TFDP3 confers MDR pattern *via* assisting cell survival upon chemotherapy-induced DNA damage. In support of this model, we designed to investigate the functional relevance of TFDP3 to MDR phenotype in MRD within childhood T-ALL.

Here, we observed that the expression of *TFDP3* increased within the MRD-H subgroup of childhood T-ALL patients after induction therapy. Preclinical experiments in mouse models further demonstrated that the selective pressure of therapy led to upregulation of TFDP3 in MRD. At the end of the 4-week induction therapy, the level of *TFDP3* was related to a 4-fold increase. Notably, the expression of TFDP3 kept at a high level in MRD during murine T-ALL relapse. Substantial DNA methylation changes have previously been reported to occur during the acquisition of chemoresistance [25, 26]. Among those changes, demethylation at associated CpG sites within promoters is thought to account for the high expression of key genes in MDR pattern [27–29]. By integrating expression profiling and promoter methylation status, we presented evidence that partially loss of promoter hypermethylation account for the high expression of TFDP3 in Jurkat/MRD lines. Taken together, induction therapy induces TFDP3 expression correlated with promoter demethylation.

In the analysis for the *in vivo* development of chemotherapeutic drug resistance activity in MRD, leukemia cells isolated from *in vivo* drug-selected xenografts were tested *in vitro* against each of the four drugs in VXLD treatment. Acquired drug resistance in Jurkat/MRD lines was identified when compared with Jurkat/E6-1 and Jurkat/Control lines. More importantly, TFDP3 silencing by RNA interference reversed MDR phenotype in Jurkat/MRD lines, indicating an important role for TFDP3 in the drug-induced DNA damage response. To understand the action of TFDP3 in MDR, we further determined whether TFDP3-mediated inhibition affected E2F1 transcriptional activity. Indeed, the base-line level of endogenous E2F1 activity decreased obviously in Jurkat/MRD lines. E2F1 is known as an inducer of apoptosis in response to various stresses especially DNA damage. E2F1-induced apoptosis is thought to contribute to the elimination of chemotherapy-injured cells that have an accumulation of DNA damage. Activation of E2F1 leads cells to apoptosis through p53- and p73-dependent pathways [30, 31]. We therefore detected the protein level of p53 and p73 in a Jurkat/MRD line following *TFDP3* downregulation. Increased expression of p53 and p73 were identified, which resulted in an accumulation of a number of proapoptotic genes, including *Bax, Puma, Noxa, Bid, Arf* and *TAp73* [16, 30]. To extend our observations *in vivo*, we tested synergy efficacy of TFDP3 silencing in combination with VXLD treatment in the Jurkat/MRD xenografts. An apparent benefit to overcome MDR in Jurkat/MRD xenografts was identified *via* intravenous injection with the lentivirus of TFDP3 shRNAs. Since TFDP3 is limited to expression in testis and most cancer tissues, including hepatocellular carcinoma, prostate cancer, breast cancer, targeting TFDP3 may provide a novel promising strategy for overcoming chemoresistance in residual cancer, and stop progression to relapse and metastatic disease.

## CONCLUSIONS

Our study demonstrates that TFDP3 contributes to MDR pattern in MRD by suppressing E2F1-induced apoptosis, and targeting TFDP3 may provide a promising strategy for improving leukemia treatment and for overcoming DNA damage based chemoresistance in residual cancer.

## MATERIALS AND METHODS

### Clinical samples

Bone marrow samples from childhood T-ALL patients at diagnosis, collected at the Hematology Laboratory of Peking University First Hospital between 2010 and 2015, were retrospectively studied. The current study was approved by the Biomedical Ethical Committee of Peking University. Mononuclear cells from patients' bone marrow were separated using the lymphocyte separation medium following the manufacturer's instructions (Lonza Walkersville Inc.). T cells were isolated from PBMC by immunomagnetic negative selection using EasySep™ Human T Cell Isolation Kit (StemCell Technologies Inc.). The purity of the isolated T cells was >95%, as determined by flow cytometric analysis. T cells from 5 healthy donors were pooled and used as an internal control [32].

### Development of Jurkat xenograft lines

All experimental procedures involving NOD/SCID mice were approved by the Biomedical Ethical Committee of Peking University. Female NOD/SCID mice aged 5 to 6 weeks were purchased from Peking University Laboratory Animal Centre (Beijing, China), and housed in a specific pathogen-free environment for 1 week prior to inoculation with human leukemia cells. Jurkat, Clone E6-1 (ATCC, Manassas, VA, USA) was expanded at 37°C in a humidified atmosphere of 5% CO_2_ and 95% air in RPMI 1640 medium (Gibco BRL) containing 10% fetal bovine serum (Gibco BRL, Grand Island, NY, USA). Jurkat xenograft NOD/SCID mice were established as previously described [21]. Engraftment and disease progression were monitored by enumerating the %huCD45^+^[20]. When the %huCD45^+^ reached 1%, mice were immediately randomized for VXLD treatment (Supplementary Table S2). A 4-week induction schedule was adopted, analogous to the clinical regimen, which delayed disease progression in all xenografts. To develop re-emerging cell lines, we stopped the VXLD treatment for 8 weeks to allow disease reappearance until %huCD45^+^ exceed 50%. Jurkat/MRD cells were purified by density gradient centrifugation, and sorted using FITC-conjugated antihuman CD45 antibody (BD, USA) on a FACSCalibur flow cytometer (BD Immunocytometry Systems, USA).

### RNA extraction and real-time quantitative PCR

Total RNA from each specimen was isolated using Trizol reagent (Life Technologies, Carlsbad, CA). RNA quality and concentration were assessed using the NanoDrop ND-1000 spectrophotometer (NanoDrop Technologies Inc.). RNA was reverse transcribed using the Superscript™ first-strand cDNA synthesis kit (TaKaRa). Fluorescence quantitative PCR instrument (Applied Biosystems 7500 Fast Real-Time PCR Systems, Life Technologies, CA, USA) and a SYBR^®^ Premix EX Tag™ kit (TaKaRa) were used to detect target gene expressions, and GAPDH was used as an internal reference. Primer sequences used in this study are described in the supporting information (Supplementary Table S3). The 2^−ΔΔCT^ method was employed to determine the relative expression of target genes normalized to GAPDH, and experiments were repeated in triplicate [33, 34].

### STRs DNA fingerprinting and methylation specific PCR

Genomic DNA was extract from Jurkat/E6-1, Jurkat/Control and Jurkat/MRD cell lines using the Gentra Puregene kit (QIAGEN). STR profiling analysis for each cell line was performed at Peking University Center for Human Disease Genomics, and compared with Jurkat/E6-1 in the ATCC databases for possible matches. The genomic DNA was also bisulfite modified and subjected to methylation-specific PCR. The primers used were 5′-ATG TTA GTT TTA TTG AAG TTA ACG A-3′ and 5′-ACT AAA CTA TCC AAA AAT TTT CGA C-3′ for the methylation reaction, and 5′-ATG TTA GTT TTA TTG AAG TTA ATG A-3′ and 5′-ACT AAA CTA TCC AAA AAT TTT CAA C-3′ for the unmethylated reaction. The PCR protocols were 40 cycles of a 30 s denaturation step at 96°C, a 30 s annealing step at 64°C, and a 30 s extension step at 72°C.

### In vitro cytotoxicity assays

Drug sensitivity was assessed using the colorimetric MTT assay as described previously [21]. Cell survival was expressed as percentage of solvent-treated controls. Results presented are the mean ± SE of at least three independent experiments.

### RNA interference targeting TFDP3

The *TFDP3* siRNA1 (5′-CAG AAG TGC TGA TGT GGA T-3′) or TFDP3 siRNA2 (OriGene, Rockville, MD, USA) was used for *TFDP3* downregulation [17]. The siRNA (5′-TTC TCC GAA CGT GTC ACG T-3′) unrelated to *TFDP3* was used as the control siRNA (Con). Non-transfected cells were used as negative control (NC). The efficiency of RNA interference on TFDP3 expression was determined by Western blotting. ShRNAs for TFDP3 siRNA1 and siRNA2 were synthesized and inserted into pLVX-shRNA vectors [35, 36]. The lentivirus of TFDP3 shRNAs was used for *in vivo* TFDP3 downregulation. To assess *in vivo* efficacy of TFDP3 shRNAs, Jurkat/MRD xenografts were treated with a 4-week VXLD, and assigned to receive intravenous either phosphate-buffered saline or non-effective shRNA (shNC) or TFDP3 shRNA1 or shRNA2 twice per week. %huCD45^+^ was monitored using flow cytometric analysis.

### Western blotting

Cells were harvested and lysed for protein extraction followed by the determination of protein concentration. The supernatant was used for Western blotting. Proteins were separated by SDS-PAGE and transferred onto polyvinylidene fluoride (PVDF) membranes. The membranes were incubated with primary antibodies, including anti-E2F1, anti-TFDP1, anti-TFDP3, anti-p53, anti-p73 and anti-β actin (Santa Cruz Biotechnology, CA, USA), followed by incubation with secondary antibodies conjugated to HRP. Signal development was performed with an ECL kit (QIAGEN). Each experiment was performed three times.

### Immunoprecipitation and immunoblotting

Cells were washed twice in phosphate-buffered saline and resuspended in lysis buffer containing 20 mM Tris (pH 7.5), 150 mM NaCl, 1% Triton X-100, 1mM EDTA, 5 μg/mL aprotinin, 5 μg/mL leupeptin, and 2mM phenylmethylsulfonyl fluoride. The lysate was incubated with anti-E2F1 antibody at a final concentration of 2 μg/mL with 25 μL of protein A-agarose (Roche Applied Science) for at least 2 h at 4°C. The precipitates were separated on polyacrylamide gels and blotted onto PVDF membranes. These bolts were then analyzed with antibodies specific for TFDP1 and TFDP3 [15].

### Luciferase assay

Cells were transfected with 0.14 μg of E2F luciferase reporter plasmid, 0.14 μg of pRL-SV140 *Renilla* luciferase reporter plasmid as an internal control, and performed as previously described [15]. The luciferase activities were determined using a dual specific luciferase assay kit (Promega). The firefly luciferase activity was normalized to the corresponding *Renilla* luciferase activity and presented as a multiple of that in cells transfected with the reporter construct alone.

### Statistical analysis

Statistical significance was determined by the two-tailed paired Student's t-test in all experiments in this study [37, 38]. The data are presented as the means ± standard deviation (SD). Values of *p*<0.05 were considered statistically significant. Asterisks indicate the statistical significance as follows: * p<0.05; ** p<0.01.

## SUPPLEMENTARY FIGURES AND TABLES


