# Supplementary material for: TFDP3 confers chemoresistance in minimal residual disease within childhood T-cell acute lymphoblastic leukemia
Source: Oncotarget. 2016 Nov 26;8(1):1405–15. doi: 10.18632/oncotarget.13630 (PMC5352064; doi:10.18632/oncotarget.13630)
Supplement: Supplementary file 1 [file oncotarget-08-1405-s001.pdf]

TFDP3 confers chemoresistance in minimal residual disease within childhood T-cell acute lymphoblastic leukemia

SUPPLEMENTARY FIGURES AND TABLES

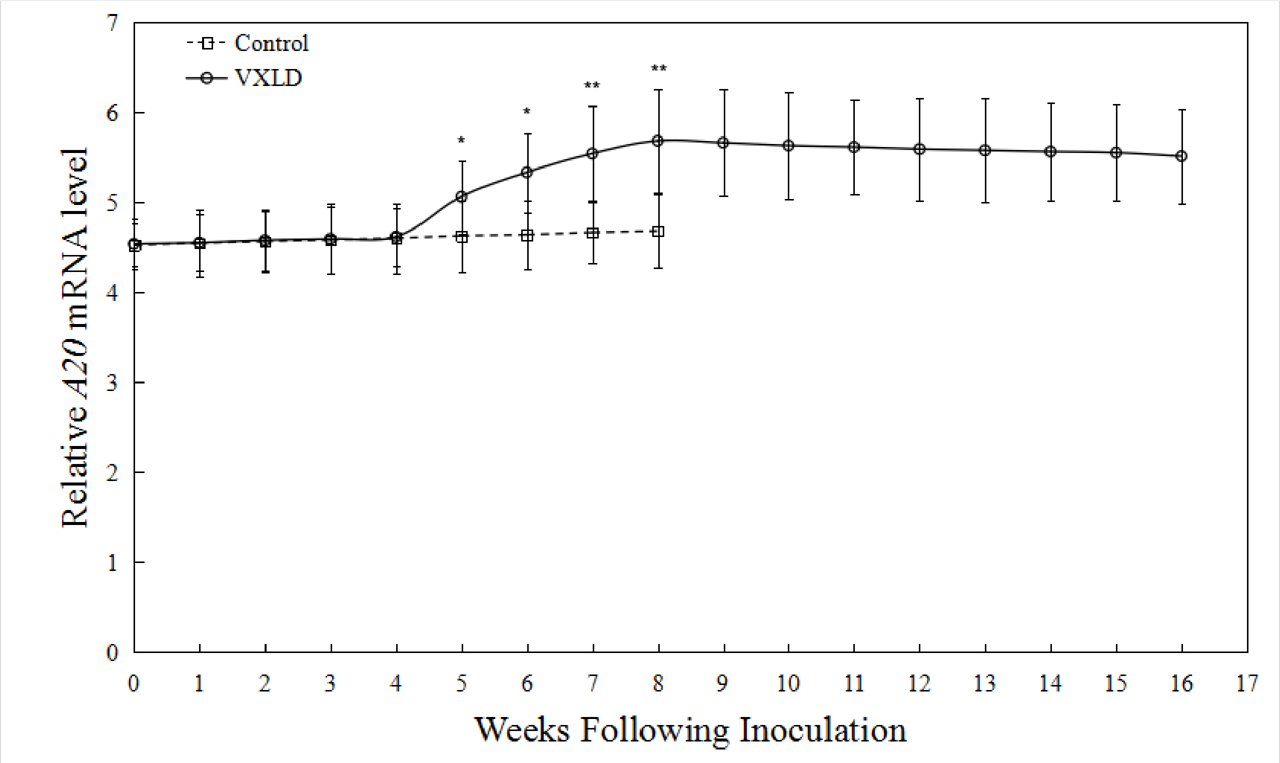

Supplementary Figure S1: The expression of *A20* during the murine T-ALL progression. *In vivo* drug treatment of Jurkat xenografts. Jurkat xenografts were treated with a 4-week induction schedule of VXLD treatment, or saline (control), and then adopted an 8-week block of VXLD to *in vivo* selection of Jurkat/MRD and Jurkat/Control lines. The expression of *A20* was assessed in the human CD45^{+} leukemia cells from murine PB. The relative *TFDP3* mRNA levels normalized to T cells from healthy donors were analyzed by real-time PCR (qPCR). Data are represented as mean±SD. ** Significance was determined at $p<0.01$ when compared with the control.

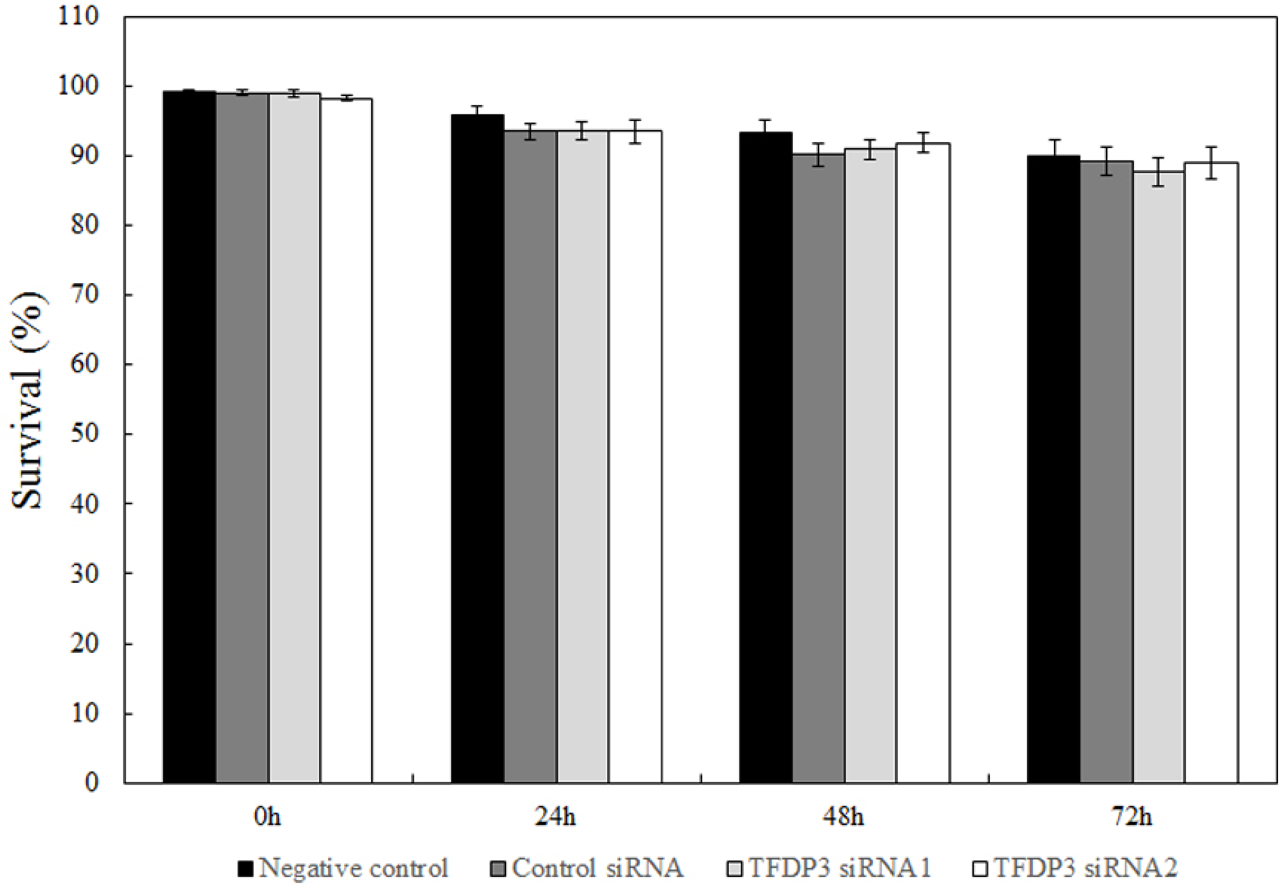

Supplementary Figure S2: Survival rate in TFDP3 knockdown Jurkat/MRD lines. Silencing of TFDP3 in Jurkat/MRD lines. Jurkat/MRD were transfected with control siRNA, *TFDP3* siRNA1 or *TFDP3* siRNA2. Non-transfection was used as negative control. Survival rate was assessed using the MTT assay at 0h, 24h, 48h, 72h. Data represent the summary of five individual Jurkat/MRD lines. Three replicate experiments were performed in each case. Data are represented as mean±SD. ** Significance was determined at $p<0.01$ when compared with the negative control.

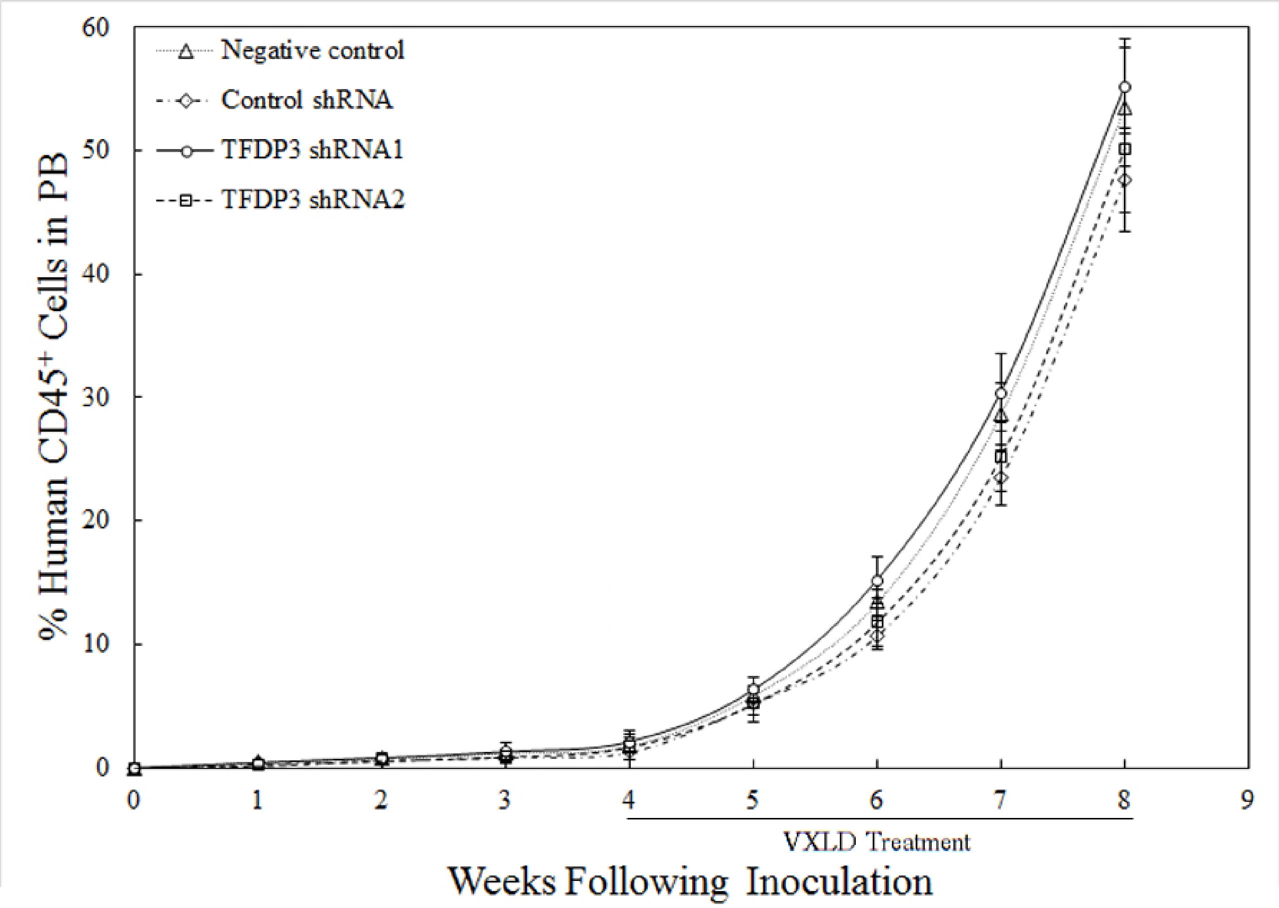

Supplementary Figure S3: Efficacy of TFDP3 silencing for Jurkat xenografts. Jurkat/MRD xenografts were assigned to receive intravenous either solvent (negative control) or non-effective shRNA (Control shRNA) or TFDP3 shRNA1 or TFDP3 shRNA2 twice per week. The %huCD45$^+$ was monitored during the treatment. ** Significance was determined at $p<0.01$ when compared with the negative control.

Supplementary Table S1: Genotyping of cell lines

Pre-clinical model	STR source	D5S818	D13S317	D7S820	D16S539	vWA	TH01	AMEL	TPOX	CSF1PO
**Control-1**	Murine $CD45^+$ cells	-	-	-	-	-	-	-	-	-
	Jurkat/ Control-1	8	8, 12	8, 12	11	18	6, 9.3	X, Y	8, 10	11, 12
**Control-2**	Murine $CD45^+$ cells	-	-	-	-	-	-	-	-	-
	Jurkat/ Control-2	8	8, 12	8, 12	11	18	6, 9.3	X, Y	8, 10	11, 12
**Control-3**	Murine $CD45^+$ cells	-	-	-	-	-	-	-	-	-
	Jurkat/ Control-3	8	8, 12	8, 12	11	18	6, 9.3	X, Y	8, 10	11, 12
**Control-4**	Murine $CD45^+$ cells	-	-	-	-	-	-	-	-	-
	Jurkat/ Control-4	8	8, 12	8, 12	11	18	6, 9.3	X, Y	8, 10	11, 12
**Control-5**	Murine $CD45^+$ cells	-	-	-	-	-	-	-	-	-
	Jurkat/ Control-5	8	8, 12	8, 12	11	18	6, 9.3	X, Y	8, 10	11, 12
**VXLD-1**	Murine $CD45^+$ cells	-	-	-	-	-	-	-	-	-
	Jurkat/MRD-1	8	8, 12	8, 12	11	18	6, 9.3	X, Y	8, 10	11, 12
**VXLD-2**	Murine $CD45^+$ cells	-	-	-	-	-	-	-	-	-
	Jurkat/MRD-2	8	8, 12	8, 12	11	18	6, 9.3	X, Y	8, 10	11, 12
**VXLD-3**	Murine $CD45^+$ cells	-	-	-	-	-	-	-	-	-
	Jurkat/MRD-3	8	8, 12	8, 12	11	18	6, 9.3	X, Y	8, 10	11, 12
**VXLD-4**	Murine $CD45^+$ cells	-	-	-	-	-	-	-	-	-
	Jurkat/MRD-4	8	8, 12	8, 12	11	18	6, 9.3	X, Y	8, 10	11, 12
**VXLD-5**	Murine $CD45^+$ cells	-	-	-	-	-	-	-	-	-
	Jurkat/MRD-5	8	8, 12	8, 12	11	18	6, 9.3	X, Y	8, 10	11, 12
	Jurkat, Clone E6-1	8	8, 12	8, 12	11	18	6, 9.3	X, Y	8, 10	11, 12

Supplementary Table S2: Development of VXLD treatment *in vivo* protocols

Drug	Action	Concentration
Vincristine	Mitotic inhibitor	0.15 mg/kg/day
Dexamethasone	Receptor mediated lympholysis	5 mg/kg/day
L-asparaginase	Asparagine depletion	1000 kU/kg/day
Daunorubicin	DNA intercalation	2.5 mg/kg/day

Supplementary Table S3: Primers for quantitative RT-PCR

Gene		Sequence
TFDP3	F	5’-ATG GAC GAG AAC CAG ACC AG-3’
	R	5’-CCC AGA CCT TCA TGG AAA GA-3’
Puma	F	5’-TGT AGA GGA GAC AGG AAT CCA CGG-3’
	R	5’-AGG CAC CTA ATT GGG CTC CAT CTC-3’
Bid	F	5’-GCA GGC CTA CCC TAG AGA CA-3’
	R	5’-GTC CAT CCC ATT TCT GGC TA-3’
Noxa	F	5’-CCA CCT GAG TTC GCA GCT CAA-3’
	R	5’-GTT GAG CAC ACT CGT CCT TCA A-3’
Bax	F	5’-TGA ACA GAT CAT GAA GAC AGG-3’
	R	5’-TGT CCA GTT CAT CTC CAA TTC-3’
TAp73	F	5’-TCG AGC ACC TGT GGA GTT C-3’
	R	5’-ACT GCT GAG CAA ATT GAA CTG-3’
Arf	F	5’-TTC TTG GTG AAG TTC GTG CGA TCC-3’
	R	5’-CGT GAA CGT TGC CCA TCA TCA TCA-3’
GAPDH	F	5’-TGA AGG TCG GAG TCA ACG GAT TTG G-3’
	R	5’-CAT GTG GGC CAT GAG GTC CAC CAC-3’
